# A systematic review of the HPV‐attributable fraction of oropharyngeal squamous cell carcinomas in Germany

**DOI:** 10.1002/cam4.2039

**Published:** 2019-03-01

**Authors:** Miriam Reuschenbach, Ingeborg Tinhofer, Claus Wittekindt, Steffen Wagner, Jens Peter Klussmann

**Affiliations:** ^1^ Department of Medical Affairs MSD Sharp & Dohme GmbH Haar Germany; ^2^ German Cancer Consortium (DKTK), Partner Site Berlin Berlin Germany; ^3^ Department of Radiation Oncology and Radiotherapy Charité University Medicine Berlin Berlin Germany; ^4^ Department of Otorhinolaryngology, Head and Neck Surgery University of Giessen Giessen Germany; ^5^ Department of Otorhinolaryngology, Head and Neck Surgery, Medical Faculty University of Cologne Cologne Germany

**Keywords:** Germany, HNC, HPV, OPSCC, prevalence

## Abstract

The prevalence of oropharyngeal squamous cell carcinoma (OPSCC) is increasing globally while the prevalence of other head and neck cancers is decreasing. The most likely reasons for this are a decreasing influence of smoking and the growing relevance of infections with the human papilloma virus (HPV) as a risk factor. A rise in the HPV‐attributable fraction (HPV‐AF) of OPSCC has been observed in many countries, yet a comprehensive review of prevalence rates and trends in Germany is lacking. To determine the current HPV‐AF of OPSCC in Germany and to assess whether it has changed during the last decades, we performed a systematic literature review. We screened Medline and EMBASE for studies that reported the tumor HPV status of newly diagnosed OPSCC patients treated at medical centers in Germany by testing for both HPV DNA and p16^INK4a^ overexpression to confirm involvement of HPV in tumorigenesis. Out of 287 screened studies, 14 studies with data from a total of 1819 OPSCC patients treated between 1988 and 2015 were included in the data synthesis. The reported average HPV‐AF varied considerably between the studies, ranging from 11.5% (1988‐2008) to 55.0% (2004‐2009). Two of the included studies did not only provide the HPV‐AF for the entire observed calendar period but also for separate years, allowing to more accurately assess changes over time. These studies reported increases in the HPV‐AF from 21% in 2000 to 53% in 2015 and from 38% in 2004 to 71% in 2013, respectively.

## 1 INTRODUCTION

### 1.1 Background

Squamous cell carcinoma of the head and neck is the sixth most common cancer worldwide and constitutes about 90% of all head and neck cancers (HNC).^1^, ^2^, ^3^ In 2008, 15 583 new HNC cases and 6100 deaths were reported in Germany alone.^2^ Oropharyngeal squamous cell carcinoma (OPSCC) is a subtype of HNC that includes cancers originating from the oropharyngeal wall and oropharyngeal subsites (palatine tonsil, base of tongue). The main risk factors for OPSCC and other HNC that have been known for a long time are excessive alcohol and tobacco consumption.^4^, ^5^, ^6^ Over the last decades, changes in risk factors and incidence trends have been observed: while the number of tobacco users and the incidence of most types of HNC are declining, the incidence of OPSCC is stagnating or even increasing in many countries, especially in developed countries.^7^, ^8^, ^9^ For example, from 1983 to 2002, a recent global study found an increasing incidence in OPSCC in several developed countries including Denmark, the Netherlands, France, Poland, and Switzerland.^10^ The most likely explanation for this increase is its causal relation with the human papilloma virus (HPV), particularly with high‐risk types such as HPV16.^10^

High‐risk HPV is a mainly sexually transmitted oncovirus that has long been known to be responsible for nearly all cervical cancer cases worldwide.^11^ A link between HPV infection and OPSCC was first suspected when case‐control studies found a strong positive correlation between HPV16 seropositivity and OPSCC.^12^, ^13^, ^14^, ^15^ Further studies that investigated the presence of HPV DNA and mRNA including the expression of the viral oncogenes E6 and E7 in the tissue of HNC corroborated these results and confirmed a causal relation between HPV and OPSCC.^9^, ^12^, ^16^ Several lines of evidence strongly suggest that HPV‐related OPSCC is a distinct tumor entity that can be distinguished from HPV‐negative OPSCC by its etiology, molecular characteristics, and clinical presentation.^17^ It became evident that patients with HPV‐related OPSCC have a significantly better response to established treatment modalities and an improved survival compared to patients with HPV‐negative OPSCC.^18^ A difference can also be observed in the affected populations: while HPV‐negative OPSCC affects primarily elderly people and heavy tobacco users, mainly younger people develop HPV‐positive OPSCC regardless of tobacco consumption, with men being affected considerably more often than women.^3^, ^9^, ^10^

Both the HPV‐attributable fraction (HPV‐AF) of OPSCC and the total incidence of OPSCC have increased in many countries during the last decades.^16^, ^19^ For example, studies with patients from Australia,^20^ Sweden,^21^ and the United States^16^ all found a strong increase in the HPV‐AF of OPSCC, especially among men and patients below the age of 60. Globally, the HPV‐AF of OPSCC increased from 7.2% in 1990‐1994 to 32.7% in 2010‐2012.^22^ These results are in‐line with recent studies from Germany, which observed a strong increase in the HPV‐AF of OPSCC and a parallel decrease in tobacco‐related OPSCC over the last years.^19^, ^23^ It is expected that by 2020, the number of HPV‐positive OPSCCs will exceed the number of cervical cancers.^16^ Thus HPV‐associated OPSCC is increasingly becoming a core HPV disease, particularly in men.

### 1.2 Rationale

No in‐depth review of the literature on the HPV‐AF of OPSCC in Germany is currently available. While a recent publication assumed an HPV‐AF for OPSCC of only 16% for estimations of disease burden in Germany,^24^ this figure is derived from an international study that reported on the average HPV‐AF for Europe in general and not for Germany in particular.^22^ Even more importantly, the same study observed strong regional differences in the HPV‐AF ranging from about 7.6% in Southern Europe to 44.9% in Central Europe. Two recent publications based on data from two German medical centers reported an HPV‐AF of more than 50% that is much closer to the figure for Central Europe than to the European average.^19^, ^23^

### 1.3 Objectives

The aim of this systematic review was to determine the most recent HPV‐AF of OPSCC in Germany and to assess whether this fraction has changed during the last decades. For this purpose, the available literature was systematically screened for prospective and retrospective studies including patients with newly diagnosed OPSCC that were treated at a German medical center. The HPV status of the tumor had to be determined by HPV DNA PCR combined with p16^INK4a^ immunohistochemistry on pretreatment tumor biopsies. The combined detection of HPV and p16^INK4a^ has demonstrated good sensitivity and specificity in a recent meta‐analysis on the detection of HPV‐driven OPSCC.^25^

## 2 METHODS

We followed the PRISMA guidelines in our literature search and in the preparation of the manuscript.^26^

### 2.1 Study eligibility criteria

The PICOTS criteria (population, intervention, comparisons, outcome, time, study design) that were used for the selection of studies eligible for inclusion into the review are shown in Table 1.

**Table 1:** PICOTS criteria for study inclusion

	Inclusion criteria
Population	At least 10 patients with newly diagnosed OPSCC (including oropharyngeal sublocations such as tonsil) and treated at a German medical center
Intervention	HPV status determined by HPV DNA PCR combined with p16^INK4a^ immunohistochemistry on pretreatment tumor biopsies
Comparisons	None
Outcome	HPV‐attributable fraction (prevalence of HPV DNA PCR and p16^INK4a^ positive OPSCC)
Time	All eligible studies will be included regardless of publication time or study duration, if they were retrievable by the searched databases by January 10, 2018
Study design	Prospective and retrospective studies including patients with newly diagnosed OPSCC without evident selection for eg stage or histology
Other	None

### 2.2 Information sources and search

A literature search was conducted in Medline and EMBASE using the search string ([HPV AND oropharynx] AND Germany) OR ([HPV AND head and neck] AND Germany). No restrictions regarding language or publication dates were applied. The last search was run on January 10, 2018. Authors with more than one included study were contacted to confirm that study populations of multiple publications from the same medical center did not overlap, and authors were asked to provide additional data for individual years if these were not reported in the published study results.

### 2.3 Study selection and data extraction

**Figure 1:**
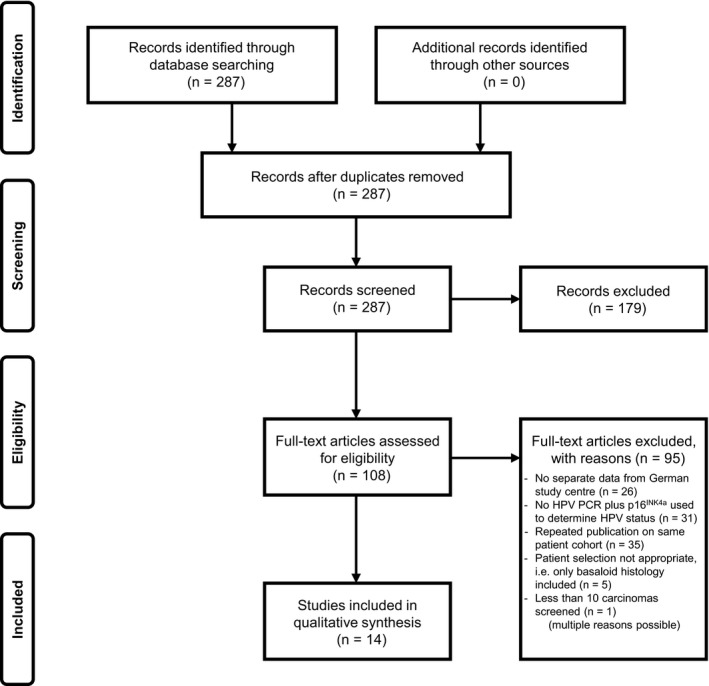
PRISMA flow diagram of the process of study selection

The process of study selection including reasons for exclusion is illustrated in the PRISMA flow diagram (Figure 1). A data extraction sheet (Table 2) was developed, according to which extraction of the data was performed by one person and validated by a second person. Extracted data include the total number of analyzed tumors, the number of tumors that were positive for both HPV DNA and p16^INK4a^ overexpression, and the following variables: study site, anatomic tumor location, case selection, calendar period of inclusion, and the type of HPV DNA PCR and p16^INK4a^ test used. Tumor stage, age, gender, tobacco use, and alcohol consumption were additionally extracted if available.

**Table 2:** HPV prevalence and methodical details of the identified studies (FF=fresh frozen, FFPE=formalin‐fixed paraffin‐embedded)

Study (year of publication) N total cohort	City	Calendar period	Case selection	OPSCC evaluable for HPV, n	OPSCC positive for HPV DNA/p16^INK4a^, n	OPSCC positive for HPV DNA/p16^INK4a^, %	HPV DNA detection method, (material, primers, read‐out)	p16^INK4a^ detection method, (material, antibody clone, positive definition)
Reimers (2007)^43^ N = 106	Cologne	1997‐2002	Consecutive patients with newly diagnosed and histologically confirmed OPSCC	96	25	26.0	FF, A5/A10 and A6/A8, electrophoresis	FFPE, 16P04, strong and diffuse cytoplasmic and nuclear staining
Hoffmann (2010)^44^ N = 39	Kiel	2004‐2007	During surgery	39	11	28.2	FF, MY09/11 and GP5/GP6, multiplex genotyping (Luminex) and Southern blot	FFPE, E6H4, strong nuclear and cytoplasmic staining,> 25%
Hoffmann (2012)^28^ N = 78	Kiel	2004‐2009	During surgery	20	11	55.0	FF, GP51/GP61 and MY09/11 plus a proprietary primer set, multiplex genotyping (Luminex) and PCR microarray	FFPE, E6H4, strong nuclear and cytoplasmic staining,> 25%
Holzinger (2012)^27^ N = 196	Heidelberg	1990‐2008	Patients diagnosed with primary OPSCC and treated at the ENT department	178	42	23.6	FF, GP5+6+,multiplex genotyping (Luminex)	TMA, E6H4 and DSC‐106, strong nuclear and cytoplasmic staining in the proliferating tumor cells
Maier (2013)^45^ N = 223	Ulm	2001‐2012	Patients treated at the ENT department	102	30	29.4	FFPE, MY09/11 plus a proprietary primer set, PCR microarray	FFPE, E6H4, strong nuclear and cytoplasmic staining
Tahtali (2013)^29^ N = 104	Frankfurt	1988‐2008	Patients with primary tumors of the oropharynx treated at the ENT department	104	12	11.5	FF, MY09/11, electrophoresis	FF, E6H4, na
Weiss (2013)^46^ N = 74	Münster	< 2013	Patients diagnosed and treated at the ENT department	59	28	47.5	FF and FFPE, GP5+6+,na	FFPE, E6H4, strong and diffuse nuclear and cytoplasmic staining
Lörincz (2014)^47^ N = 35	Hamburg	2011‐2013	Patients selected for TORS	35	12	34.3	na, na, na	na, na, na
Meyer (2014)^48^ N = 106	Cologne	2000‐2005	Curative resection at ENT department	93	25	26.9	FF and FFPE, A5/A10 and A6/A8, electrophoresis	FFPE, 16P04, strong nuclear and cytoplasmic staining
Quabius (2014)^49^ N = 36	Kiel	2012	During surgery at the ENT department	19	5	26.3	FF, MY09/11 and GP51/GP61, electrophoresis and Sanger sequencing	FFPE, E6H4, 30%, moderate 31‐75% and strong> 75%
Tinhofer (2015)^19^ N = 227	Berlin	2004‐2013	Patients who had been treated at the radiation oncology department within the last 10 years	227	91	40.1	na, GP5+6+, multiplex genotyping (Luminex)	na, E6H4, strong nuclear staining in> 70% cells
Hauck (2015)^31^ N = 424	Berlin	1997‐2011	Selected from pathology, according to availability of paraffin blocks	122	42	34.4	FFPE, GP5+6+, electrophoresis and Sanger sequencing	FFPE, E6H4, > 70% of neoplastic cells strong nuclear and/or cytoplasmic staining
Hoffmann (2018)^30^ N = 126	Kiel	2002‐2010	During panendoscopy or surgery at the ENT	126	48	38.1	FFPE, MY09/11 and GP51/GP61, electrophoresis and Sanger sequencing	FFPE, E6H4, strong diffuse staining
Würdemann (2017)^23^ N = 599	Giessen	2000‐2015	Patients diagnosed between 01/01/2000 and 07/15/2016 and sufficient tumor material available	599	150	25.0	FFPE, GP5+6+, multiplex genotyping (Luminex)	FFPE, E6H4, diffuse staining

### 2.4 Risk of bias in individual and across studies

Only studies that tested tumors for both HPV DNA and for p16^INK4a^ overexpression to indicate an actual involvement of HPV in tumorigenesis were included. Testing only for the presence of HPV DNA or overexpression of p16^INK4a^ was considered insufficient. Additionally, we only included studies that reported results for the OPSCC cases separately from other HNC cases, that included patients at the time of diagnosis or treatment without evident selection for stage or histology, which provided separate data for a German medical center if an international cohort was analyzed and that tested tumors from at least 10 individual patients. To reduce bias resulting from studies with overlapping cohorts, we only included the most recent or most comprehensive publication from centers that repeatedly reported on the same patient population.

## 3 RESULTS

### 3.1 Characteristics of included studies

Out of 287 screened studies, 14 fulfilled the inclusion criteria and were included in the data synthesis. These 14 studies covered a total of 1819 individual OPSCC patients with no overlap between cohorts. A PRISMA flow diagram of the process of study selection is presented in Figure 1.

All included studies report the HPV‐AF of OPSCC in newly diagnosed patients that were treated at medical centers in Germany, using both HPV DNA PCR and p16^INK4a^ immunohistochemistry to determine the HPV status of the tumor tissue. The studies cover varying time periods from 1988 to 2015 as shown in Table 2. The primary outcome obtained from all studies was the HPV‐AF of OPSCC. Secondary outcomes could be obtained from some of the included studies, however, often not separately for the OPSCC cases included in this review but for the entire studied HNC cohorts. These were tumor sub‐site (6/14 studies), tumor stage (12/14), age (all 14), gender, (all 14), tobacco use (8/14), and alcohol consumption (6/14). A summary of these results can be found in Table S1.

### 3.2 Reported HPV‐attributable fraction

**Figure 2:**
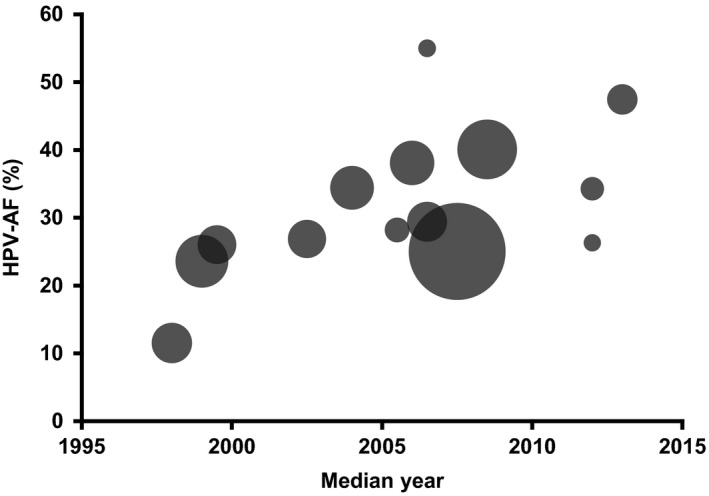
Bubble plot of the reported HPV‐AF of OPSCC as a function of the median year of the observed time period. Each bubble represents an individual study. Bubble area corresponds to the number of patients included in the study, ranging from 19 patients (smallest bubble) to 599 patients (largest bubble)

The reported overall HPV‐AF varied considerably between the individual studies, ranging from 11.5%^29^ to 55.0%^28^ (Table 2). The HPV‐AF reported by the individual studies as a function of the median year of the observed time period is presented in Figure 2.

**Figure 3:**
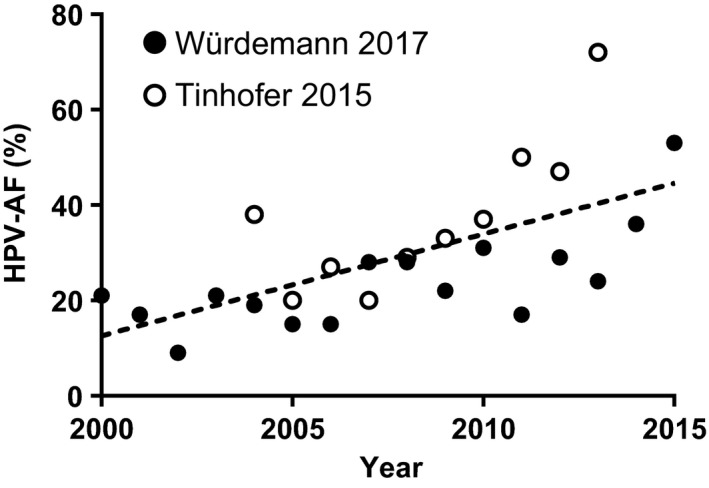
Changes in the HPV‐AF of OPSCC in two German cohorts observed between 2004‐2013 (empty circles)^19^ and 2000‐2015 (filled circles).^23^ The dashed trend line was derived by linear regression

Two of the included studies did not only provide the HPV‐AF for the entire cohort of the observed period but also for separate years, allowing to more accurately assess the change in the HPV‐AF over time (Figure 3). One study (from Giessen) covering the years from 2000 to 2015 reported an HPV‐AF of 21% in 2000 and of 53% in 2015, with an average increase of 1.6% per year.^23^ The second study (from Berlin) covering the years from 2004 to 2013 obtained similar results, with an HPV‐AF of 38% in 2004 and 72% in 2013.^19^

### 3.3 Risk of bias within and across studies

**Figure 4:**
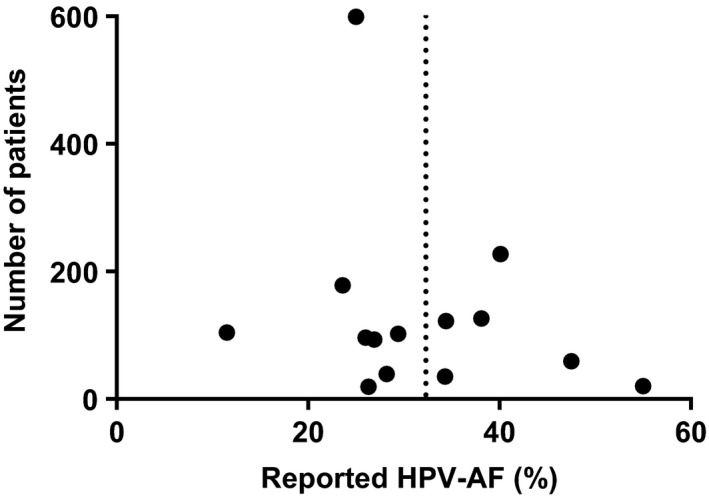
Funnel plot of study precision (number of included patients) vs primary outcome (HPV‐AF). The dotted line indicates the mean value

To reduce the risk of bias in individual studies, we excluded studies that did not meet the inclusion requirements as shown in Figure 1 *.* To assess the risk of bias across the included studies, a funnel plot was used (Figure 4). The reported HPV‐AF was considered the primary outcome, and the number of included patients was used as a measure of study precision.

The distribution of the studies around the mean in the funnel plot showed no obvious asymmetry that might indicate a possible bias.

Since the reported HPV‐AF might artificially differ between studies that used HPV detection methods with different sensitivities, we tested for this potential bias by comparing the HPV‐AF reported by studies using different detection methods. To test for significance, we used either Student's *t* test (two parameters) or one‐way analysis of variance with Tukey's test (three or more parameters). Out of the 14 studies included into the data synthesis, 13 reported the DNA‐ and p16^INK4a^‐based detection method they used (Table 2). No significant difference was found between different materials, primers, or read‐outs in the case of DNA‐based methods or between different tissue fixation or antibodies in the case of p16^INK4a^‐based methods (Table S2). Taken together, no apparent bias based on differences in detection methods could be found.

## 4 DISCUSSION

### 4.1 Summary of evidence

This systematic literature review demonstrates that the published HPV‐AF of OPSCC in Germany varies strongly between different studies, ranging from 11.5%^29^ to 55.0%.^28^ The two included studies that provided the HPV‐AF for several successive years both observed a marked increase over time. For the most recent years they covered, 2013 and 2015, they reported a respective HPV‐AF of 72% and 53%.^19^, ^23^ It can thus be assumed that the HPV‐AF of OPSCC has increased strongly in Germany during the last decades and is currently above 50%.

### 4.2 Limitations

There are three major limitations that need to be considered when interpreting the data summarized in this review. The first limitation is related to the process of patient selection in the individual studies and insufficient definition and inconsistency in the published reports concerning anatomical tumor locations in the head and neck region. We therefore restricted the analysis to OPSCC in order to focus on the anatomic location most strongly associated with HPV. Studies were excluded if patient selection appeared not appropriate (ie if only basaloid histology was included). Still we were not able to separate the results for oropharyngeal sublocations, which might be important to account for the higher HPV‐AF known for tumors located at the palatine tonsils compared to base of tongue and other sites.

The second limitation of data on HPV‐AF is related to the method used to determine the HPV status, which is likely to contribute to heterogeneity between the individual studies. We only included studies that determined the HPV status by combining HPV PCR with p16^INK4a^ immunohistochemistry to minimize this bias. Still, HPV PCR techniques are poorly harmonized and interpretation of p16^INK4a^ immunohistochemistry can be heterogeneous. While testing for viral E6/E7 mRNA expression is a reliable alternative to testing for HPV association in OPSCC, it is less commonly used and still lacks standardization. It has been demonstrated that the combination of HPV DNA plus p16^INK4a^ yields results comparable to E6/E7 mRNA testing.^25^

The third limitation is related to the varying calendar periods addressed in the studies. Since it is assumed that the HPV‐AF increased over the last decades and thus is influenced by the time period of diagnosis, data synthesis for several calendar periods would be desirable. However, the observation periods varied strongly between the individual studies, and only two studies report changes of prevalence over time. It was thus decided to restrict data synthesis and to report the prevalence of HPV in the individual studies without performing a meta‐analysis.

### 4.3 Comparison of the current HPV‐AF with previous studies

In a large international study from 2016, the HPV‐AF of OPSCC in Europe was reported to be 16% if determined by HPV DNA, p16^INK4a^ and HPV mRNA, and 19.1% if determined by HPV DNA and p16^INK4a^ as in the present review.^22^ This figure was presumed to be representative of the situation in Germany by some following studies.^24^ However, the vast majority of the samples on which the study is based were obtained before 2010, and several recent publications based on data from medical centers in Germany reported considerably higher figures for the HPV‐AF of OPSCC.^19^, ^23^, ^30^, ^31^ To address this discrepancy, the present study reviewed the available literature on the HPV‐AF of OPSCC in Germany.

The same study that calculated the average European HPV‐AF of OPSCC to be 19.1% also reported four distinct HPV‐AF values for different geographic regions of Europe as defined by the GLOBOCAN classification.^32^ These were 25% for Northern Europe (and up to about 50% with most test methods) and 45.7% for Central and Eastern Europe, yet only 18.1% for Western Europe and 7.7% for Southern Europe.^22^ However, a majority of 67.5% of the European cancer samples tested for HPV in this study was obtained from centers in Southern Europe, whereas the fractions of samples from Northern, Western, and Central/Eastern Europe were only 6.5%, 8.9%, and 17.1%, respectively. Since the total European figure was not corrected for these highly unequal regional contributions, it reflects mainly the situation in Southern Europe and not in Europe as a whole. A generalization of this figure to Europe in general or to individual European countries thus neglects the clear difference in the prevalence of HPV‐attributable OPSCC between northeastern and southwestern European countries that was reported by this study. If the four regions are weighed according to their respective population at the time of publication,^33^ the figure for the total European HPV‐AF of OPSCC rises to 27.8%, which is considerably higher and probably closer to the real percentage than the reported 19.1%. A recent study on the HPV‐AF of cancer cases in Germany draws on this figure, although the authors acknowledge that prevalence varies strongly between different countries and that a generalization of the European figure is thus likely an under‐ or overestimation.^24^ In particular, they point toward two recent studies which reported an HPV‐AF for the German OPSCC population that was much higher than the European estimate.^19^, ^34^ The results of the present systematic review support this notion since the most recent studies reviewed herein all report an HPV‐AF of OPSCC that is much higher than 19.1%.

### 4.4 Comparison of changes in the HPV‐AF with previous studies

Concerning a possible change in the HPV‐AF of OPSCC over time, our results indicate a strong increase during the last decades, particularly based on the results of two studies by Tinhofer et al and Würdemann et al.^19^, ^23^ In these two studies, HPV‐AF was determined individually for separate time periods (2004‐2006 to 2012‐2013) and years (2000 to 2015), respectively. Both studies concluded that there was a strong and highly significant increase in the HPV‐AF (by 115% and 152%) in the observed periods. Several studies show a similar trend for other countries such as the Netherlands,^35^ Denmark,^36^ Sweden,^21^ and the United States.^9^, ^16^ While we could not identify a systematic review on the HPV‐AF of OPSCC that focused on any individual European country, a recent systematic review and meta‐analysis of the literature on global trends in the prevalence of HPV in OPSCC reports a strong, significant increase from pre‐1995 to 2015 both globally and in Europe.^37^ That review included eight studies, which specifically reported on the situation in Germany and performed a meta‐analysis of all included studies with a median year of the sample collection period after 2000. In this meta‐analysis, the prevalence of HPV in OPSCC in Germany was calculated to be 46% (95% CI 36%‐56%). However, the presence of viral DNA in a tumor sample cannot be considered a precise surrogate for an actual etiological involvement of the virus in tumorigenesis. Since HPV DNA PCR alone was the only test for HPV performed in the studies included in that meta‐analysis, only the fraction of HPV‐positive tumors could be assessed, which is not necessarily the same as the HPV‐AF. In fact, more recent publications have shown that additional testing for either E6/E7 mRNA or p16^INK4a^ expression is necessary to assume an actual involvement of HPV in tumorigenesis of OPSCC.^12^ Therefore, there was no overlap between the studies included in the review by Stein et al and the present review. It is accepted that testing for additional parameters provides a more robust picture of the actual HPV‐AF of OPSCC. A clear advantage of the present review is the requirement for p16^INK4a^ expression testing in addition to HPV DNA among included studies. The combined analysis of p16^INK4a^ plus HPV DNA has been demonstrated to accurately identify HPV E6/E7‐expressing OPSCC.^25^

While the reported increase in the HPV‐AF of OPSCC in Germany fits into the general observation of an increase in developed countries worldwide, the reasons for this increase remain unclear. Part of a possible explanation is the fact that the prevalence of tobacco use, which is a major risk factor for HPV‐negative OPSCC, has decreased markedly in developed countries since the 1980s (from 33.1% in 1980 to 23.5% in 2012), that is in parallel to the increase in HPV‐positive OPSCC.^3^, ^38^ This global trend is also reflected by the situation in Germany, where a decrease in the prevalence of tobacco use from 33.2% in 1980 to 25.0% in 2012^38^ parallels the increase in the HPV‐AF of OPSCC observed in the present study. One might thus assume that a relative increase in the HPV‐AF can be attributed to decreasing tobacco use alone. However, since high‐risk HPV is mainly sexually transmitted, it is assumed that changes in sexual behavior in developed countries during the last decades also play a role in the increase in the HPV‐AF of OPSCC.^9^ An increasing number of lifetime sexual partners, increasing prevalence of oral sex, and younger age at first sexual contact are considered to be the major contributors to this trend.^3^, ^12^, ^39^ A recent systematic review of studies on sexual behavior as a risk factor for OPSCC found that a high number of sexual partners and performing oral sex were the two factors most strongly associated with a greater risk of OPSCC.^40^ In 2017, a survey of 2524 persons representative of the German population estimated the mean lifetime number of sexual partners of the opposite sex to be 10.23 for men and 5.46 for women. Regarding the prevalence of active oral sex, 51% of men and 45% of women interviewed reported to have engaged in this practice at least once in their life.^41^ Since this was the first ever assessment of sexual behavior in Germany based on a representative sample, no historical data are available for comparison. However, trends in behavior can be inferred from the data for different age groups obtained in this study. For example, about 75% of men and 68% of women in the age group of 24‐29 years reported to have performed oral sex on a partner at least once in their life, whereas these percentages were considerably lower in the age groups above 50 years, for example 43% and 36% in the age group of 60‐69 years and 24% and 17% in the age group of 80‐100 years, respectively. Since the reported prevalence of ever having engaged in vaginal intercourse was about equally high (88%‐98%) among all adult age groups, this data can be interpreted as evidence that the prevalence of oral sex has indeed increased in Germany since the middle of the 20th century. This assumption matches data from other countries such as the United States, where a similar trend has been reported based on historical data.^37^ In 2014, a study concluded that differences in oral sexual behavior alone may sufficiently explain epidemiological differences in oral infection with the OPSCC high‐risk strain HPV16 across gender, age, and race.^42^ This study reported that men had both a higher mean number of total sexual partners (18.4 vs 7.8) and of partners they performed oral sex on (9.9 vs 3.8) than women, which the authors conclude to be responsible for the higher prevalence of oral HPV infection among men (11.4% vs 3.3%). In general, the lifetime number of oral sex partners has been established as the factor most strongly associated with OPSCC.^39^

## 5 CONCLUSION

The majority of studies included in the present review report an HPV‐AF of OPSCC in Germany that is considerably higher than the previously assumed 16.0%. This is especially true for studies covering recent time periods, which report HPV‐AF values of up to 55%.^28^ Comparing the reported HPV‐AF with the observed time periods strongly indicates that the HPV‐AF of OPSCC in Germany has increased notably over the last decades. Since it is assumed that there is a time lag of about 20‐30 years between initial HPV infection and cancer development,^37^ a person that was exposed to HPV at age 25 would develop cancer at age 45‐55. Considering that the prevalence of performing oral sex has strongly increased in recent generations and is highest in individuals now aged 24‐29 years,^41^ it can be assumed that the incidence of HPV‐attributable OPSCC will continue to rise.

## CONFLICT OF INTEREST

Miriam Reuschenbach is an employee of MSD Sharp & Dohme GmbH and MSD Sharp & Dohme GmbH Germany provided funding for this work. Jens Peter Klussmann received a research grant from MSD Sharp & Dohme GmbH Germany, and Claus Wittekindt and Steffen Wagner are participants of this granted research project. Ingeborg Tinhofer has nothing to disclose.
