# Supplementary material for: A systematic review of the HPV‐attributable fraction of oropharyngeal squamous cell carcinomas in Germany
Source: Cancer Med. 2019 Mar 1;8(4):1908–18. doi: 10.1002/cam4.2039 (PMC6488137; doi:10.1002/cam4.2039)
Supplement: Supplementary file 1 [file CAM4-8-1908-s001.docx]

**Table S1: Secondary outcome data from the included studies.**

| Outcome | **Included Studies** | | | | | | |
| --- | --- | --- | --- | --- | --- | --- | --- |
|  | **Reimers**  **(2007)**  **N = 106** | **Hoffmann**  **(2010)**  **N = 39** | **Hoffmann**  **(2012)**  **N = 78** | **Holzinger**  **(2012)**  **N = 196** | **Maier**  **(2013)**  **N = 223** | **Tahtali**  **(2013)**  **N = 104** | **Weiss**  **(2013)**  **N = 74** |
| **Gender, n (%)** | | | | | | | |
| Male | 83 (78.0) | 32 (82.1) | 66 (84.6) | 146 (74.0) | 188 (84.3) | 87 (83.7) | 49 (66.2) |
| Female | 23 (22.0) | 7 (17.9) | 12 (15.4) | 50 (26.0) | 35 (15.7) | 17 (16.3) | 25 (33.8) |
| **Age (Years)** | | | | | | | |
| Mean (SD) | 59.0 (43.0–80.0) | 60.2 (51.5–68.9) | 60.0 (51.6–68.5) | n.a. | 58.4 (n.a.)^f^ | 58.2 (n.a.) | 63.5 (10.7) |
| Median (Range) | n.a. | 60 (42–84) | 59 (43–79) | 57 (n.a.) | n.a. | 58 (n.a.) | n.a. (43–89) |
| **Tumor site, n (%)** | | | | | | | |
| Oropharynx | 106 (100.0) | 39 (100) | 20 (25.6) | 196 (100.0) | 102 (45.7) | 104 (100.0) | 59 (79.7) |
| Tonsils | 63 (59.4) | 39 (100) | 20 (25.6) | 84 (43.0) | 56 (25.1) | n.a. | n.a. |
| Base of tongue | 14 (13.2) | 0 | 0 | 45 (23.0) | 46 (20.6) | n.a. | n.a. |
| Other | 29 (27.4) | 0 | 0 | 67 (34.0) |  | n.a. | n.a. |
| Oral cavity | 0 | 0 | 12 (15.4) | 0 | 24 (10.8) | 0 | 11 (14.9) |
| Hypopharynx | 0 | 0 | 24 (30.8) | 0 | 55 (24.7) | 0 | 1 (1.4) |
| Larynx | 0 | 0 | 22 (28.2) | 0 | 42 (18.8) | 0 | 3 (4.1) |
| **T stage^a^, n (%)** | | | | | | | |
| T1 | n.a. | 3 (7.7) | 0 | 87 (44.4)^c^ | n.a. | 12 (11.5) | 44 (59.5) |
| T2 | n.a. | 14 (35.9) | 7 (9.0) |  | n.a. | 37 (35.6) |  |
| T3 | n.a. | 14 (35.9) | 15 (19.2) | 107 (54.6)^c^ | n.a. | 16 (15.4) | 24 (32.4) |
| T4 | n.a. | 8 (20.5) | 22 (28.2) |  | n.a. | 39 (37.5) |  |
| Missing | n.a. | 0 | 34 (43.6) | 2 (1.0)^c^ | n.a. | 0 | 6 (8.1) |
| **N stage^a^, n (%)** | | | | | | | |
| N0 | 32 (30.2)^b^ | 13 (33.3) | 29 (37.2) | 47 (24.0)^c^ | n.a. | 28 (26.9) | 40 (54.1) |
| N1 | 74 (69.8)^b^ | 6 (15.4) | 9 (11.5) | 147 (75.0)^c^ | n.a. | 15 (14.4) |  |
| N2 |  | 18 (46.2) | 36 (46.2) |  | n.a. | 47 (45.2) | 27 (36.5) |
| N3 |  | 2 (5.1) | 4 (5.1) |  | n.a. | 11 (10.6) |  |
| Nx | n.a. | 0 | 0 | 0^c^ | n.a. | 3 (2.9) |  |
| Missing | n.a. | 0 | 0 | 2 (1.0)^c^ | n.a. | 0 | 7 (9.5) |
| **M stage^a^, n (%)** | | | | | | | |
| M0 | n.a. | 38 (97.4) | 69 (88.5) | 179 (91.3)^c^ | n.a. | n.a. | 65 (87.8) |
| M1 | n.a. | 1 (2.6) | 5 (6.4) | 9 (4.6)^c^ | n.a. | n.a. | 4 (5.4) |
| Missing | n.a. | 0 | 4 (5.1) | 8 (4.1)^c^ | n.a. | n.a. | 5 (6.8) |
| **UICC stage^a^ , n (%)** | | | | | | | |
| I | 4 (3.8)^b^ | 0 | 4 (5.1) | 60 (30.6)^c^ | 25 (11.2) | 6 (5.8) | 18 (24.3) |
| II | 15 (14.2) ^b^ | 24 (61.5) | 59 (75.6)^i^ |  | 21 (9.4) | 15 (14.4) |  |
| III | 18 (17.0) ^b^ | 10 (25.6) | 15 (19.2) |  | 37 (16.6) | 17 (16.3) | 49 (66.2) |
| IV | 69 (65.1) ^b^ | 0 | 0 | 134 (68.4)^c^ | 119 (53.4) | 66 (63.5) |  |
| Unknown/Missing | 0 | 5 (12.8) | 0 | 2 (1.0)^c^ | 21 (9.4) | 0 | 7 (9.5) |
| **Smoking, n (%)** | | | | | | | |
| yes | 89 (84.0) | n.a. | n.a. | 173 (88.3)^d^ | 143 (64.4)^g^ | n.a. | 39 (52.7) |
| no | 18 (17.0) | n.a. | n.a. | 21 (10.7)^d^ | 66 (29.8)^g^ | n.a. | 33 (44.6) |
| Unknown/Missing | 0 | n.a. | n.a. | 2 (1.0) | 14 (6.3)^g^ | n.a. | 2 (2.7) |
| **Alcohol, n (%)** | | | | | | | |
| yes | 72 (67.9) | n.a. | n.a. | 178 (90.8)^e^ | 103 (46.2)^h^ | n.a. | 33 (44.6) |
| no | 25 (23.6) | n.a. | n.a. | 15 (7.7)^e^ | 106 (47.5)^h^ | n.a. | 39 (52.7) |
| Unknown/Missing | 0 | n.a. | n.a. | 3 (1.5) | 14 (6.3) | n.a. | 2 (2.7) |
| a: Tumour staging was assessed according to the UICC criteria (7^th^ edition).  b: Tumour staging was assessed according to the AJCC criteria (6^th^ edition).  c: Unclear which tumour staging criteria were used.  d: Yes (former or current) vs. No (never).  e: Yes (regular alcohol consumption) vs. No (moderate or no alcohol consumption).  f: Calculated by information given in the publication.  g: Yes (higher consumption) vs. No (no regular or moderate alcohol consumption).  h: Yes (regular alcohol consumption) vs. No (no alcohol consumption).  i: Three tumours classified as stage 2–3 in the original study were included under stage 2 in this table.  j: Yes (≥ 2 standard drinks) vs. No (< 2 standard drinks).  k: Data from the German cohort was used. The data from the Brazilian cohort is available in the publication.  m: Defined as N+.  n: Data available but not compatible with classification: 90 patients (71.4 %) consumed more than 10 packs of cigarettes per year, 36 patients (28.6 %) consumed less. | | | | | | | |

| Outcome | **Included Studies** | | | | | | |
| --- | --- | --- | --- | --- | --- | --- | --- |
|  | **Lörincz**  **(2014)**  **N = 35** | **Meyer**  **(2014)**  **N = 106** | **Quabius**  **(2014)**  **N =36** | **Tinhofer**  **(2015)**  **N = 436** | **Hauck**  **(2015)**  **N = 424^k^** | **Hoffmann**  **(2018)**  **N = 126** | **Würdemann**  **(2017)**  **N = 599** |
| **Gender, n (%)** | | | | | | | |
| Male | 26 (74.3) | 75 (70.8) | 26 (72.2) | 359 (82.3) | 322 (68.3) | 91 (77.2) | 466 (77.8) |
| Female | 9 (25.7) | 31 (29.2) | 10 (27.8) | 77 (17.7) | 102 (31.7) | 35 (27.8) | 133 (22.2) |
| **Age (years)** | | | | | | | |
| Mean (SD) | n.a. | n.a. | 64.9 (11.3) | n.a. | n.a. | n.a. | n.a. |
| Median (range) | 65 (49–84) | 57 (34–79) | 64.5 (45.0‑87.0) | 58.6 (29–86) | 60 (41–92) | 59.9 (36.3–88.9) | 59.7 (36.9–91.8) |
| **Tumour site, n (%)** | | | | | | | |
| Oropharynx | 35 (100.0) | 106 (100) | 19 (52.8) | 227 (52.1) | 124 (29.2) | n.a. | 599 (100.0) |
| Tonsils | 13 (37.1) | n.a. | 13 (36.1) | n.a. | n.a. | n.a. | n.a. |
| Base of tongue | 14 (40.0) | n.a.  n.a. | 6 (16.7) | n.a. | n.a. | n.a. | n.a. |
| Other | 8 (22.9) |  |  | n.a. | n.a. | n.a. | n.a. |
| Oral cavity | 0 | 0 | 3 (8.3) | 64 (14.7) | 265 (62,5) | n.a. | 0 |
| Hypopharynx | 0 | 0 | 6 (16.7) | 116 (26.6) | 35 (8,3) | n.a. | 0 |
| Larynx | 0 | 0 | 8 (22.2) | 13 (3.0) | 0 | n.a. | 0 |
| **T-Stage^a^, n (%)** | | | | | | | |
| T1 | 19 (54.3) | 29 (27.4) | n.a. | n.a. | 287 (67.7) | n.a. | n.a. |
| T2 | 15 (42.9) | 29 (27.4) | n.a. | n.a. |  | n.a. | n.a. |
| T3 | 1 (2.8) | 16 (15.1) | n.a. | n.a. | 117 (27.6) | n.a. | n.a. |
| T4 |  | 30 (28.3) | n.a. | n.a. |  | n.a. | n.a. |
| Missing | 0 | 2 (1.9) | n.a. | n.a. | 20 (4.7) | n.a. | n.a. |
| **N-Stage^a^, n (%)** | | | | | | | |
| N0 | 13 (37.1) | 16 (15.8) | n.a. | n.a. | 165 (38.9) | n.a. | n.a. |
| N1 | 8 (22.9) | 21 (20.8) | n.a. | n.a. | 224 (52.8)^m^ | n.a. | n.a. |
| N2 | 13 (37.1) | 49 (48.5) | n.a. | n.a. |  | n.a. | n.a. |
| N3 | 1 (2.9) | 15 (14.9) | n.a. | n.a. |  | n.a. | n.a. |
| Nx | 0 | n.a. | n.a. | n.a. | 0 | n.a. | n.a. |
| Missing | 0 | n.a. | n.a. | n.a. | 35 (8.3) | n.a. | n.a. |
| **M-Stage^a^, n (%)** | | | | | | | |
| M0 | n.a. | 82 (77.4) | n.a. | n.a. | n.a. | n.a. | n.a. |
| M1 | n.a. | 7 (6.6) | n.a. | n.a. | n.a. | n.a. | n.a. |
| MX |  | 14 (13.2) | n.a. | n.a. | n.a. | n.a. |  |
| Missing | n.a. | 3 (2.8) | n.a. | n.a. | n.a. | n.a. | n.a. |
| **UICC-Stage^a^ , n (%)** | | | | | | | |
| I | 13 (37.1) | n.a. | n.a. | 0 | n.a. | n.a. | 62 (10.4) |
| II |  | n.a. | n.a. | 6 (1.4) | n.a. | n.a. | 52 (8.7) |
| III | 22 (62.9) | n.a. | n.a. | 40 (9.2) | n.a. | n.a. | 99 (16.5) |
| IV |  | n.a. | n.a. | 390 (89.4) | n.a. | n.a. | 386 (64.4) |
| Unknown/Missing | 0 | n.a. | n.a. | 0 | n.a. | n.a. | 0 |
| **Smoking, n (%)** | | | | | | | |
| yes | n.a. | 73 (68.9) | 20 (55.6) | 286 (65.6) | n.a. | n.a.^n^ | 121 (20.2) |
| no | n.a. | 10 (9.4) | 16 (44.4) | 150 (34.4) | n.a. | n.a.^n^ | 261 (77.0) |
| Unknown/Missing | n.a. | 23 (21.7) | 0 | 0 | n.a. | n.a.^n^ | 17 (2.8) |
| **Alcohol, n (%)** | | | | | | | |
| yes | n.a. | 72 (67.9) | n.a. | n.a. | n.a. | n.a. | 288 (48.1)^j^ |
| no | n.a. | 11 (10.4) | n.a. | n.a. | n.a. | n.a. | 288 (48.1)^j^ |
| Unknown/Missing | n.a. | 23 (21.7) | n.a. | n.a. | n.a. | n.a. | 23 (3.8) |
| a: Tumour staging was assessed according to the UICC criteria (7^th^ edition).  b: Tumour staging was assessed according to the AJCC criteria (6^th^ edition).  c: Unclear which tumour staging criteria were used.  d: Yes (former or current) vs. No (never).  e: Yes (regular alcohol consumption) vs. No (moderate or no alcohol consumption).  f: Calculated by information given in the publication.  g: Yes (more consumption) vs. No (no regular or moderate alcohol consumption).  h: Yes (regular alcohol consumption) vs. No (no alcohol consumption).  i: Three tumours classified as stage 2–3 in the original study were included under stage 2 in this table.  j: Yes (≥ 2 standard drinks) vs. No (< 2 standard drinks).  k: Data from the German cohort was used. The data from the Brazilian cohort is available in the publication.  m: Defined as N+.  n: Data available but not compatible with classification: 90 patients (71.4 %) consumed more than 10 packs of cigarettes per year, 36 patients (28.6 %) consumed less. | | | | | | | |

**Table S2: Comparison of methods used for HPV status assessment of tumour tissue in regard to a possible effect on the reported HPV-AF values.**

| Methods | **Mean HPV-AF ± SD** | ***p* value** | **Significance** |
| --- | --- | --- | --- |
| **DNA-based methods compared by raw material** | | | |
| FF vs. FFPE | 30.6±13.0 vs. 33.6±7.6 | 0.658 | n.s. |
| **DNA-based methods compared by primers** | | | |
| MY09/11 vs. GP5+6+ | 26.7±8.6 vs. 35.4±10.2 | 0.304 | n.s. |
| MY09/11 vs. A10/A5-A6/A8 | 26.7±8.6 vs. 26.5±0.4 | 1.000 | n.s. |
| GP5+6+ vs. A10/A5-A6/A8 | 35.4±10.2 vs. 26.5±0.4 | 0.512 | n.s. |
| **DNA-based methods compared by read-out** | | | |
| Multiplex genotyping vs. electrophoresis | 34.4±11.8 vs. 27.2±8.4 | 0.826 | n.s. |
| Multiplex genotyping vs. Sanger sequencing | 34.4±11.8 vs. 32.9±4.9 | 1.000 | n.s. |
| Multiplex genotyping vs. PCR microarray | 34.4±11.8 vs. 42.2±12.8 | 0.916 | n.s. |
| Multiplex genotyping vs. Southern blotting | 34.4±11.8 vs. 28.2 | 0.986 | n.s. |
| Electrophoresis vs. Sanger sequencing | 27.2±8.4 vs. 32.9±4.9 | 0.948 | n.s. |
| Electrophoresis vs. PCR microarray | 27.2±8.4 vs. 42.2±12.8 | 0.506 | n.s. |
| Electrophoresis vs. Southern blotting | 27.2±8.4 vs. 28.2 | 1.000 | n.s. |
| Sanger sequencing vs. PCR microarray | 32.9±4.9 vs. 42.2±12.8 | 0.891 | n.s. |
| Sanger sequencing vs. Southern blotting | 32.9±4.9 vs. 28.2 | 0.996 | n.s. |
| PCR microarray vs. Southern blotting | 42.2±12.8 vs. 28.2 | 0.844 | n.s. |
| **p16^INK4a^-based methods compared by raw material** | | | |
| FFPE vs. FF | 33.7±9.7 vs. 11.5 | 0.154 | n.s. |
| FFPE vs. TMA | 33.7±9.7 vs. 23.6 | 0.633 | n.s. |
| FF vs. TMA | 11.5 vs. 23.6 | 0.693 | n.s. |
| **p16^INK4a^-based methods compared by antibody** | | | |
| E6H4 vs. 16P04 | 32.6±11.5 vs. 26.5±0.4 | 0.500 | n.s. |
